# Increased summer temperature is associated with reduced calf mass of a circumpolar large mammal through direct thermoregulatory and indirect, food quality, pathways

**DOI:** 10.1007/s00442-023-05367-0

**Published:** 2023-04-05

**Authors:** Sheila M. Holmes, Sabrina Dressel, Julien Morel, Robert Spitzer, John P. Ball, Göran Ericsson, Navinder J. Singh, Fredrik Widemo, Joris P. G. M. Cromsigt, Kjell Danell

**Affiliations:** 1 Department of Wildlife, Fish and Environmental Studies, Swedish University of Agricultural Sciences, 90183 Umeå, Sweden; 2 Forest and Nature Conservation Policy Chair Group, Wageningen, The Netherlands; 3 Department of Agricultural Research for Northern Sweden, Swedish University of Agricultural Sciences, 90183 Umeå, Sweden

**Keywords:** *Alces alces*, Climate change impacts on mammals, Direct effects, Indirect effects, Sweden

## Abstract

Climate change represents a growing ecological challenge. The (sub) arctic and boreal regions of the world experience the most rapid warming, presenting an excellent model system for studying how climate change affects mammals. Moose (*Alces alces*) are a particularly relevant model species with their circumpolar range. Population declines across the southern edge of this range are linked to rising temperatures. Using a long-term dataset (1988–1997, 2017–2019), we examine the relative strength of direct (thermoregulatory costs) and indirect (food quality) pathways linking temperature, precipitation, and the quality of two important food items (birch and fireweed) to variation in moose calf mass in northern Sweden. The direct effects of temperature consistently showed stronger relationships to moose calf mass than did the indirect effects. The proportion of growing season days where the temperature exceeded a 20 °C threshold showed stronger direct negative relationships to moose calf mass than did mean temperature values. Finally, while annual forb (fireweed) quality was more strongly influenced by temperature and precipitation than were perennial (birch) leaves, this did not translate into a stronger relationship to moose calf weight. The only indirect path with supporting evidence suggested that mean growing season temperatures were positively associated with neutral detergent fiber, which was, in turn, negatively associated with calf mass. While indirect impacts of climate change deserve further investigation, it is important to recognize the large direct impacts of temperature on cold-adapted species.

**Supplementary Information:**

The online version contains supplementary material available at 10.1007/s00442-023-05367-0.

## Introduction

Global climate change is a growing ecological challenge. By 2020, global temperatures had increased by approximately 1.09 °C over pre-industrial levels (IPCC 2021). Additionally, we are already experiencing more frequent and intense weather extremes (droughts, heavy precipitation events, hot days and nights), and variability in weather across years is predicted to increase with further climate change (IPCC 2021). Such changes impact many species, with some shifting poleward, and others experiencing increased risk of extinction (IPCC 2021). Temperature increases may affect animals directly by adding thermoregulatory costs. With temperatures more often above their thermal tolerances, individuals may be at risk of lethal hyperthermia or may incur direct energetic costs through physiological or behavioural thermoregulation (Fuller et al. 2016; Hetem et al. 2014; McKechnie and Wolf 2019). Increased temperatures may also affect animals indirectly via phenological mismatches between animal life history events and the phenology of their food (Plard et al. 2014; Post and Forchhammer 2008), and key interactions between co-occurring species (Beard et al. 2019; Cohen et al. 2018). Such mismatches may lead to reduced energy intake for consumers, or even whole-ecosystem consequences, for example, in nutrient cycling (Beard et al. 2019).

Climate change is increasingly seen as a major threat to global large mammal populations (Fuller et al. 2016; Rondinini and Visconti 2015). Mammal species from the (sub) arctic and boreal regions of the world present an excellent model system for studies on climate warming and mammal fitness, because those regions experience the fastest warming. Of the (sub) arctic and boreal mammals, the moose (*Alces alces*) is a large circumpolar ungulate that shows signs of potential vulnerability to climate change. This species is experiencing population declines at the southern edge of its range (Dou et al. 2013; Monteith et al. 2015; Murray et al. 2006; Schrempp et al. 2019). These declines are largely attributed to direct and indirect effects of rising temperatures, and are predicted to continue (Rempel 2011; Weiskopf et al. 2019), but the primary proximate factors vary across studies. These include endoparasites and infectious disease (Murray et al. 2006), blood loss and anemia from winter tick (*Dermacentor albipictus*) infestation (Jones et al. 2019), availability of high-quality forage (Monteith et al. 2015; Schrempp et al. 2019), and thermoregulatory costs affecting energy balance (Dou et al. 2013; Monteith et al. 2015). Factors may vary across sites, or in some cases interact; for example poor nutrition or energy balance may leave individuals susceptible to parasites or disease (Murray et al. 2006).

In Sweden, declines in recruitment and calf mass, particularly in the southern counties, have been linked to hot, dry spring weather, suggesting a similar effect of climate change on these Eurasian moose as seen in earlier studies on the North American subspecies (Holmes et al. 2021). While European moose lack the negative impacts of the winter tick, studies have indicated potentially substantial direct thermoregulatory costs, as well as indirect effects via the nutritional quality of forage.

### Direct effects

Moose are cold adapted, with lower upper critical temperature and heat stress points than other northern ungulates (Parker and Robbins 1984; Renecker and Hudson 1986). Moose metabolic, respiration, and heart rates all increase around 14–20 °C, with open-mouthed panting beginning at temperatures above 20 °C (McCann et al. 2013; Renecker and Hudson 1986, 1990), though wind and shade may mitigate temperature effects, increasing upper critical temperatures (McCann et al. 2013). Direct costs may extend beyond metabolic expenditure, however, as food intake is known to drop with higher ambient temperatures in many taxa (Youngentob et al. 2021). Reduced food intake decreases thermogenesis due to digestion (Youngentob et al. 2021). Moose have also been known to reduce travel (Thompson et al. 2021) and increase resting at high ambient temperatures (Ditmer et al. 2018) and to increase use of thermal shelters (e.g., mature coniferous forest, wet areas; Verzuh et al. 2021; Verzuh et al. 2022). The selection of some of these thermal shelters, mature coniferous forest particularly, may reduce access to forage, particularly at temperatures over 20 °C (Van Beest et al. 2012; Verzuh et al. 2021, 2022). Despite the potential foraging costs of behavioral thermoregulation, adult females that optimize use of foraging habitats and thermal shelters, based on ambient temperature, tend to gain more summer mass (Van Beest and Milner 2013).

### Indirect effects

Growing season length (green-up to freeze) and low soil nitrogen (N) availability tend to limit primary productivity in the boreal forest (Jarvis and Linder 2000; Price et al. 2013). Early spring marks both the highest quality vegetation and the energetically expensive lactation period for adult female moose (Neumann et al. 2020). Synchronizing lactation with peak vegetation quality is important for offspring survival and calf weight gain in ungulates (Parker et al. 2009; Plard et al. 2014). At higher spring/summer temperatures, green-up occurs earlier and/or faster (Doi and Katano 2008; Douhard et al. 2018; Pettorelli et al. 2005, 2007). This may mean that peak vegetation quality occurs earlier in spring (Doi and Katano 2008; Douhard et al. 2018; Pettorelli et al. 2005), before parturition and the lactation period when energy and nutrients are most needed (Neumann et al. 2020). This could have a negative effect on moose calf weight gain. Spring precipitation may interact with high temperatures to speed up plant phenology and increase productivity earlier in the season (Loison et al. 1999; Pettorelli et al. 2007; Wu et al. 2011). However, such increased productivity associated with higher levels of plant-available moisture may also result in lower plant nutrient content (Olff et al. 2002). Conversely, higher temperatures in conjunction with low precipitation levels reduce plant productivity, likely due to water stress (Hoeppner and Dukes 2012; Wu et al. 2011). Climate change could, thus, lead to both an earlier peak in vegetation quality and/or reduced vegetation quality, both negatively affecting lactation and calf weight gain.

With food quality playing a potentially important role in the effects of climate change on moose performance, indicators of food quality become important. Elemental N has been suggested as a useful food quality indicator, particularly for nitrogen-limited environments such as the boreal forest (Rizzuto et al. 2021), where consumers like moose may also select food on the basis of N concentration (Ball et al. 2000). Plant digestibility tends to increase with N concentration and decrease with higher concentrations of fiber and secondary metabolites (Forsyth et al. 2005; McArt et al. 2009; Spalinger et al. 2010). For several common broadleaved moose forage species, N and crude protein peak rapidly in the spring and then decline as fiber and secondary compounds start to increase (Capoani 2019; McArt et al. 2009; Shively et al. 2019). Therefore, earlier phenology is associated with earlier reduction in plant digestibility and protein (Albon and Langvatn 1992; Hebblewhite et al. 2008). Indeed, higher average temperatures in spring and summer have been associated with lower plant N but greater tannin or phenolic content (Bø and Hjeljord 1991; Lenart et al. 2002). Low water availability likewise results in lower plant protein content, in addition to lower biomass (Deléglise et al. 2015; White et al. 2014).

Both quantity and quality of forage can affect herbivore growth and survival. At low biomass levels, energy intake tends to be limited by forage quantity, but at intermediate biomass, it is more limited by digestible energy and protein content (Hebblewhite et al. 2008; Parker et al. 2009). Indeed, small increases in forage quality can have large consequences for the mass and reproduction of ungulates (White 1983). Elk (*Cervus elaphus*) show reduced mass loss in winter with increased N intake (Christianson and Creel 2009), and a diet supplemented with N led to greater subcutaneous fat depth in muskoxen (Peltier and Barboza 2003). Moose appear to maximize N intake in their diet; they preferentially use and browse more on N-fertilized trees, though it is important to note that N-fertilization also increased forage quantity (Ball et al. 2000; Månsson et al. 2009). Female moose intake follows N and fiber patterns, peaking in green-up or late spring, and declining with increasing temperatures and acid detergent fiber (Shively et al. 2019). Among ungulates, offspring weight is a good indicator of population fitness, as young are highly susceptible to environmental conditions and early growth has lasting repercussions for survival to adulthood as well as adult condition and reproductive success (Albon et al. 1987; Festa-Bianchet et al. 1997; Gaillard et al. 2003; Keech et al. 2011; Parker et al. 2009). Rapid green-up has been associated with less growth and, in some cases, survival between lamb/kid and yearling stages in alpine ungulates, suggesting a greater impact of forage quality than availability on offspring growth and survival (Pettorelli et al. 2007). Similarly, climatic factors associated with higher forage quality were also linked to greater body mass and calf growth in Norwegian moose (Herfindal et al. 2006).

### Objectives

We combined three closely linked objectives to understand the consequences of climate change on moose. First, to determine the degree to which increasing temperatures and/or changing precipitation patterns affect moose calf mass indirectly, through effects on forage quality, or directly (Q1), we compared the strength of direct and indirect pathways using piecewise structural equation modelling (SEM). However, not all aspects of temperature change at equal rates. With climate change, the frequency of weather extremes is expected to increase even faster than climatic averages (Beniston et al. 2007; Meehl et al. 2000). As different aspects of temperature may influence moose differently (Holmes et al. 2021), it is important to determine if direct and/or indirect effects vary if we look at changes in average temperature or changes in extreme temperature events. For our second objective, we, therefore, examined if the number of days with temperatures reaching or exceeding 20 °C has different indirect and direct relationships to moose calf mass than average temperature (Q2). Finally, temperature increase tends to have a larger effect on the advance of spring phenology in annuals than in perennials (Stuble et al. 2021). Our third objective, therefore, asked if we, as expected, see a stronger impact of weather on the quality of annuals (forbs) than perennials (woody species), and if this variation in quality then better predicts calf mass variation (Q3).

## Methods

### Vegetation

#### Collection and sample preparation

**Fig. 1:**
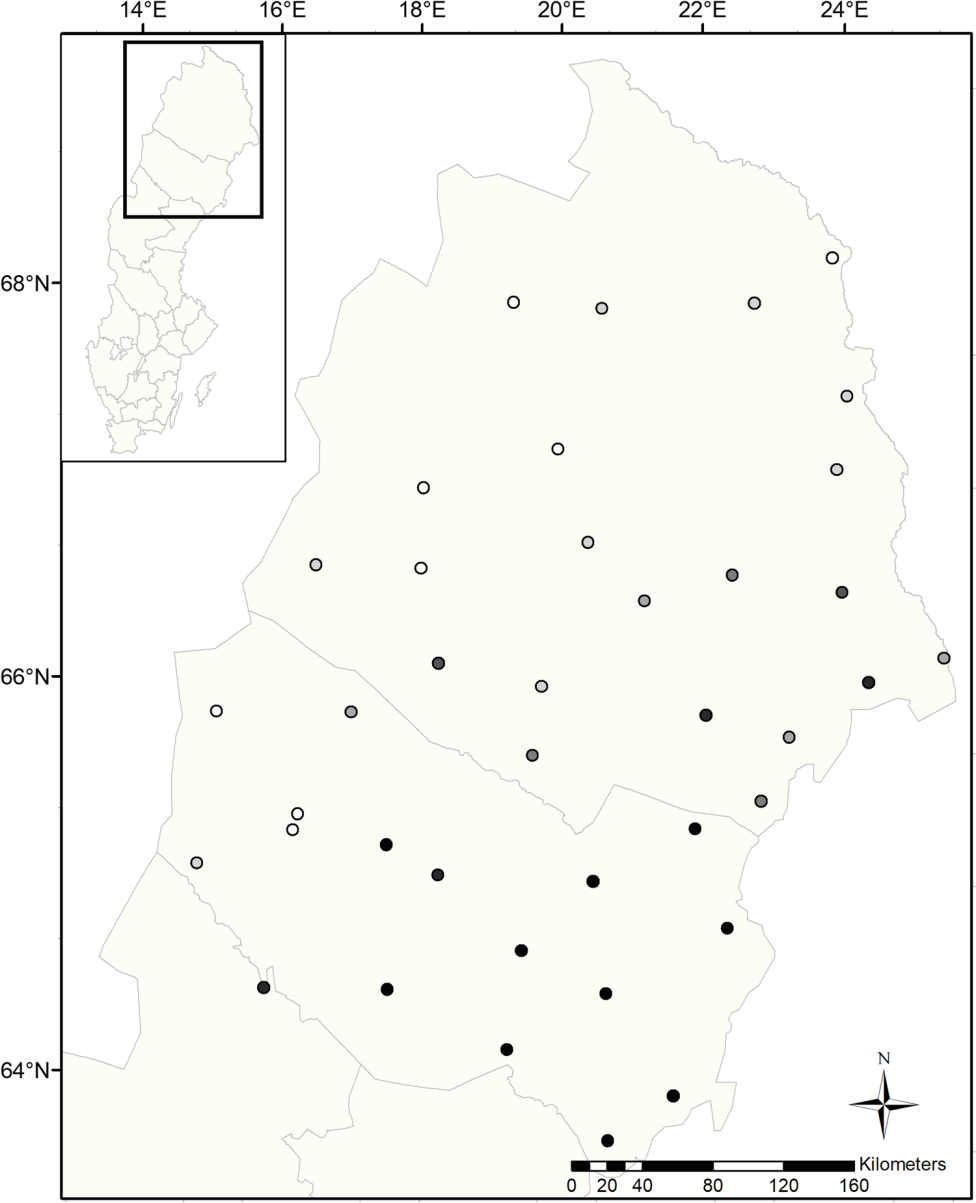
Map of study area in northern Sweden with vegetation sampling locations indicated. Circle shading represents the number of years a data point is included in the final models (see Methods for more information on restrictions). N ranges from 0 (white) to 13 (black)

We collected vegetation samples at 13 to 39 sites per year across Norrbotten and Västerbotten counties, northern Sweden, from 1988 to 1997 and 2017 to 2019 (Fig. 1). We visited sites between July 17 and 25 each year, collecting two important summer diet plants for moose: one annual forb, fireweed (*Epilobium angustifolium,* synonym *Chamerion angustifolium*), and one woody perennial, downy birch (*Betula pubescens*) (Cederlund et al. 1980; Sæther et al. 1996). We sampled in the middle of the fireweed flowering period to detect variation in phenology better across sites and years. Each site consisted of a 1 ha sampling location in a moose-preferred foraging habitat (e.g., near a young forest, bypass or secondary road). We visited sites as close to the same date as possible across years. If site conditions changed significantly between sampling years (e.g., scarified by forestry), we established a new site, as close to the original site as possible.

From each site, we collected stalks and/or leaves from 30 individuals each of fireweed and birch, simulating moose summer browsing. We selected a representative sample with the same proportion of individuals budding or flowering as in the full 1 ha area, and sampling covered the full 1 ha area. Sampled birch trees were approximately 2–2.5 m tall, and we stripped leaves from the ends of branches at three locations per tree—at approximately 2–2.5 m, 1.5 m, and 1 m height. Samples were taken from different aspects of the tree to correct for potential sun effects on food quality. We clipped fireweed stalks at the base. We combined all samples of a given species into one paper bag at each site. If the total sample volume exceeded 1.5 L, we cut stalks into approximately 10 cm-long sections over a tray, mixed samples thoroughly together by hand, and selected a random 1.5 L sample.

Within zero to 4 days of collection, we placed sample bags, open, in drying cabinets at 40 °C. We stirred samples at least once per day to encourage faster and more even drying. When samples were completely dry, we milled birch leaves on a Cyclotec^™^ 1093 hammer mill (Foss Analytical AB). We pre-ground fireweed stalks on either a Kamas (Kamas, Malmö, Sweden; 1988–1997) or a Retsch SM 300 cutting mill (Haan, Germany; 2017–2019), followed by a Cyclotec™ 1093 mill. We used a screen diameter of 1 mm for all milling.

#### NIRS data acquisition

Milled samples were scanned with a Specim SWIR 3 hyperspectral camera. Acquired hypercubes consisted of 384 pixels width and variable length images with a spatial resolution of approximately 0.31 mm and 288 spectral bands ranging between 1000 and 2495 nm. The samples were illuminated using two rows of halogen lamps with an angle of approximately 45° relative to the nadir axis. For each sample, the reflected light was converted to reflectance using a Spectralon for the white reference and shutter closing for the dark reference. Hyperspectral images were then processed to extract samples-related pixels for further analyses.

#### NIRS modeling

To reduce the costs linked to traditional wet chemistry analyses, a PCA-selected subset of samples (i.e., that were representative of the spectral variability of the complete dataset) was sent to Dairy One Forage Laboratory, USA, for dry matter, neutral detergent fiber (NDF), and nitrogen (N) contents analyses (*n* = 50). The *pls* function of the pls R library (Liland et al. 2021) was used to adjust partial least-square regression models (hereinafter referred to as PLSR) based on a leave-one-out cross validation approach and using the spectral bands as the explanatory variables and the laboratory-measured traits as the variables to explain. Following the standard procedure of the pls library to avoid scale effects, data were mean centered before adjusting the models. Estimation performances of PLSR models were evaluated using the relative root mean square error ($RMSE$) and the coefficient of determination (${R}^{2}$). Developed models were eventually used to estimate dry matter, neutral detergent fiber, and nitrogen contents for the complete dataset.

### Weather

We obtained daily weather records, including minimum, maximum, and mean temperature as well as total precipitation, from the Swedish Meteorological and Hydrological Institute (SMHI, www.smhi.se/). Using ArcGIS (version 10.5), we used the Point Distance tool to determine all weather stations within 50 km of each vegetation sampling location (site). This represented the shortest radius that allowed for all sites to have both temperature and precipitation data for the full study period. On average, there were approximately 18 stations within 50 km of each site with a mean distance of 32 km. Stations varied in the years they were active and the weather metrics recorded.

We followed SMHI’s definition of the start of the growing season as the first day of the first four consecutive days each calendar year that each have a mean daily temperature greater than or equal to 5 °C. We did this separately for each station that measured temperature and then took the earliest growing season start date for all stations within 50 km of a site to represent the start of the growing season for that site. We calculated all weather variables between that date and July 17 each year.

For each weather station, we calculated the total precipitation, mean temperature, and the proportion of days where the maximum temperature reached or exceeded 20 °C. We then averaged the temperature and precipitation variables across all weather stations within the 50 km radius of each site.

### Moose calf weights

#### Data collection

We provided weighing scales to and sent data collection templates to hunting teams across Västerbotten and Norrbotten counties and asked them to measure calf slaughter weight for moose calves shot from September to December yearly during 1988 to 1997 (supplementary Table 1S). For each moose calf shot, teams recorded the date, sex, and slaughter weight (kg; as per Langvatn 1977) of each calf, as well as the grid cell location on a map to indicate where the calf was shot. Slaughter weight represented total body mass minus skin, viscera, lower legs, head, and blood (as per Langvatn 1977). We then assigned each grid cell a GPS point, representing the center point of the cell. Sample size per year between 1988 and 1997 ranged from 991 to 1680 (total *n* = 12,747 moose) with an overall sex ratio of approximately 1.07 males per female calf sampled.

For 2017–2020, we used publicly available data on moose slaughter weights (identical definition as above) voluntarily reported by hunters in the official management database, Älgdata (www.algdata.se). This database is run by the 20 Swedish County Administrative Boards managing moose. We extracted moose calf slaughter weights from Älgdata for the period from September 2017 to January 2020. Each year, moose calf weights would represent calves shot during the hunting season September–January. To obtain spatial data with sufficient precision to match vegetation and weather data, we used only moose for which GPS coordinates had been reported.

#### Growth slope adjustment

We matched moose calf weights to the vegetation and weather characteristics of the nearest vegetation sampling site up to a maximum distance of 50 km (matching the radius for weather variables). Of 3976 calf weights reported within 50 km of vegetation plots in 2017–2019, the average distance from plots was approximately 26.8 km. We used a conservative cutoff for realistic calf weights, accounting for errors in reporting of weights, by omitting those less than 10 kg or more than 120 kg. We then adjusted for September growth of moose calves (there was no evidence of gain or loss of weight after October 1, see Holmes et al. 2021) by adjusting all weights of September-shot calves to October 1 based on county-, year-, and sex-specific regression coefficients, as in Holmes et al. (2021). If the number of individuals per county, year, and sex grouping was below ten individuals, we omitted calves from further analyses to avoid biased weight adjustments due to low sample size. We then joined moose calf weights to the vegetation and weather data, omitting years and sites for which no vegetation was collected. The full dataset included 15,056 calf weights. We then calculated the mean calf weights per year and site, omitting year x site combinations with less than ten calves to reduce the risk of biasing model results due to extremely low sample sizes at some sites. This left a total of 236 site by year combinations.

### Models

We used confirmatory path analysis to explore the relative strength of the direct and indirect relationships between weather, vegetation quality, and moose calf mass. Path analysis is a special form of structural equation modeling (SEM) which simultaneously calculates several regression models. Thereby, variables can act as dependent variables in one part of the model and as independent variables in others. This allows the calculation of indirect and direct effects between variables. Given the data structure, sample size, and distribution of several variables, we chose piecewise SEM, as it allows for flexibility in model structure, permitting inclusion of hierarchical structure, interactions, random effects, and correlation structures (Lefcheck 2016; Shipley 2009).

We used four separate models to address our three main research questions. The four models varied across two factors: species (fireweed vs. birch) and temperature measurement (average temperature vs. proportion of days with a maximum temperature ≥ 20 °C). These models allowed us to see the effect of the temperature metric used, and the type of food plant, on the relationship between weather and calf mass.

**Fig. 2:**
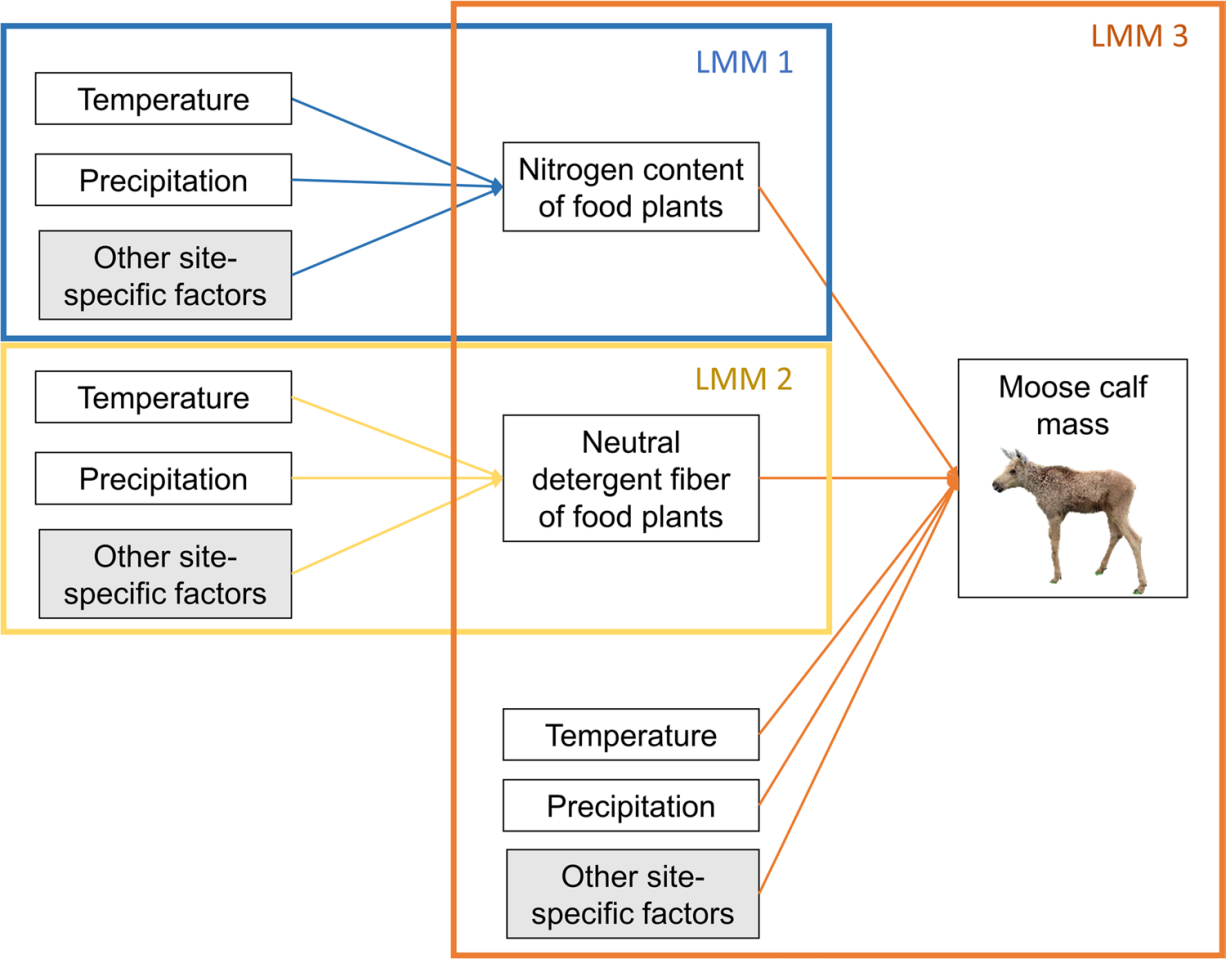
Conceptual model of the three linear mixed models (LMM) that make up each of the structural equation models. Each LMM is encapsulated by a different colored box, and uses different colored arrows. Arrows lead from predictor variables to response variables. Random factors are shaded. Note that the temperature, precipitation, and random factors were the same for all three LMMs within each SEM

For each piecewise SEM, we used the function ‘psem’ from the R package ‘piecewiseSEM’ (Lefcheck 2016) in combination with ‘lme’ from the package ‘nlme’ (Pinheiro et al. 2022) to connect three separate linear mixed models (LMM). The first two models included total precipitation and a temperature measurement as predictors and NDF or N for either birch or fireweed as the response variable (Fig. 2, LMMs 1 and 2). The third model included total precipitation, temperature, and NDF and N as predictor variables and mean moose calf mass as the response (Fig. 2, LMM 3). All LMMs accounted for spatial and temporal autocorrelation and differences in unmeasured factors across locations by including site as a random factor, along with a continuous AR(1) correlation structure. Given the previously recorded inverse pattern of N and NDF over the growing season (Shively et al. 2019), we also included a correlative relationship between NDF and N.

As we applied SEM in a confirmatory way to test our assumed theoretical model, no model modifications were carried out. We evaluated the model fit via the strength and significance of involved path coefficients and the retained R^2^ values.

## Results

### Changes in average summer temperature, proportion of days > 20 degrees, and calf mass

Despite some inter-annual variability, we detected no long-term changes in mean summer temperature ($\overline{x }$ = 10.7 °C SD = 1.28), proportion of days > 20 °C ($\overline{x }$ = 0.2, SD = 0.1), and mean calf mass ($\overline{x }$ = 70.0 kg, SD = 4.8) across the study sites for years included in the study (Fig. 1S).

### Direct and indirect relationships between weather, vegetation quality, and moose calf mass

PLSR models showed acceptable to good prediction accuracies, with ${R}^{2}$ of 0.63, 0.69, and 0.88 for dry matter, neutral detergent fiber, and nitrogen contents, respectively. In terms of $RMSE$, satisfactory performances were obtained for all three variables ($RMSE$ of 0.37, 2.67, and 0.16%, for DM, NDF, and N contents, respectively).

**Fig. 3:**
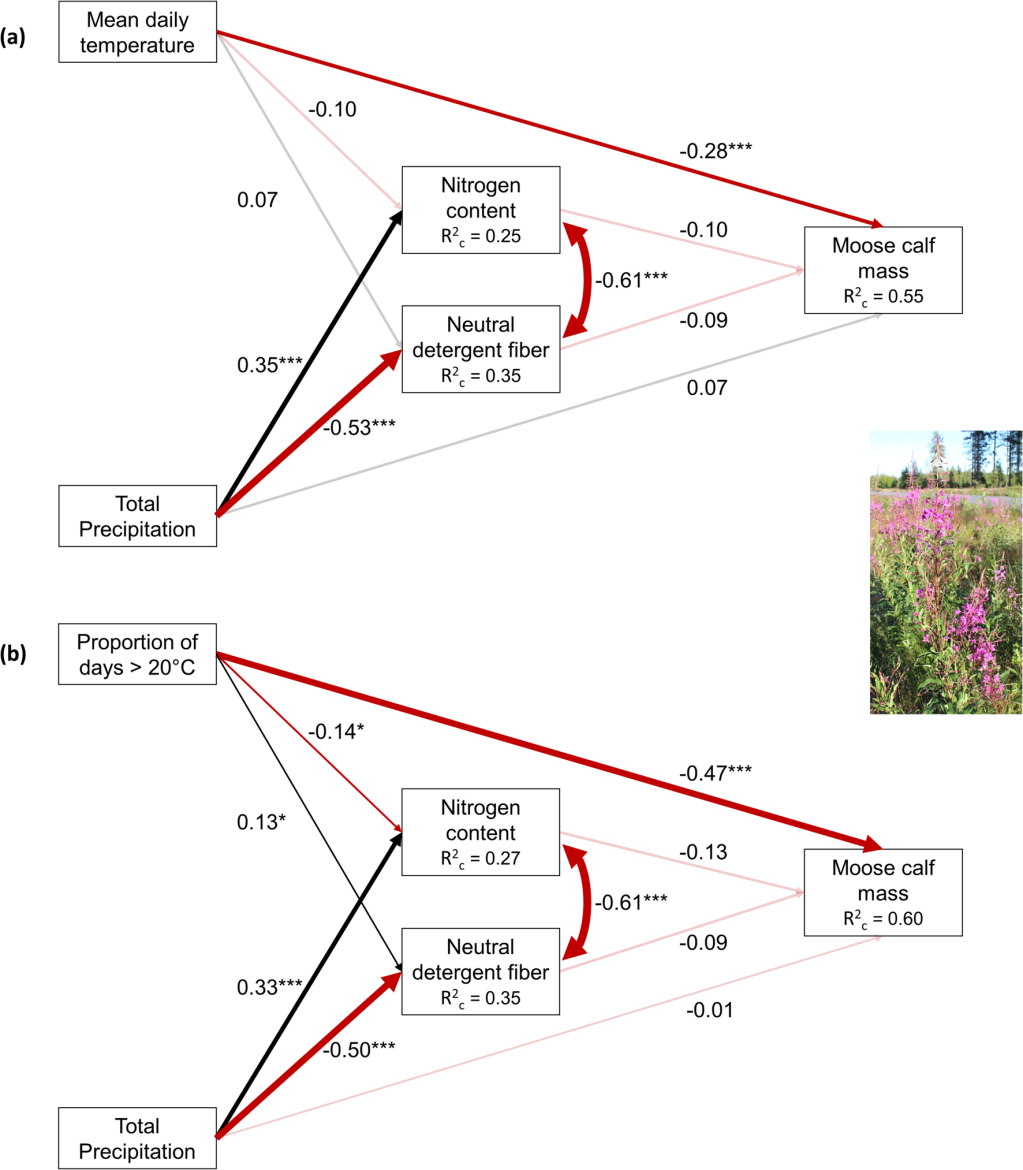
Results of the SEM comparing the direct relationship between weather, including **a** mean temperature and **b** proportion of days > 20 °C, and moose calf mass to the indirect relationship via nitrogen and neutral detergent fiber of fireweed stems, leaves, and flowers. Black lines represent positive relationships, red lines represent negative relationships. Line thickness is proportional to effect size. Transparent lines represent relationships with a *p* value more than the 0.05 significance level. R^2^_c_ values represent the variance of each dependent variable that is explained by fixed and random factors within that LMM

**Fig. 4:**
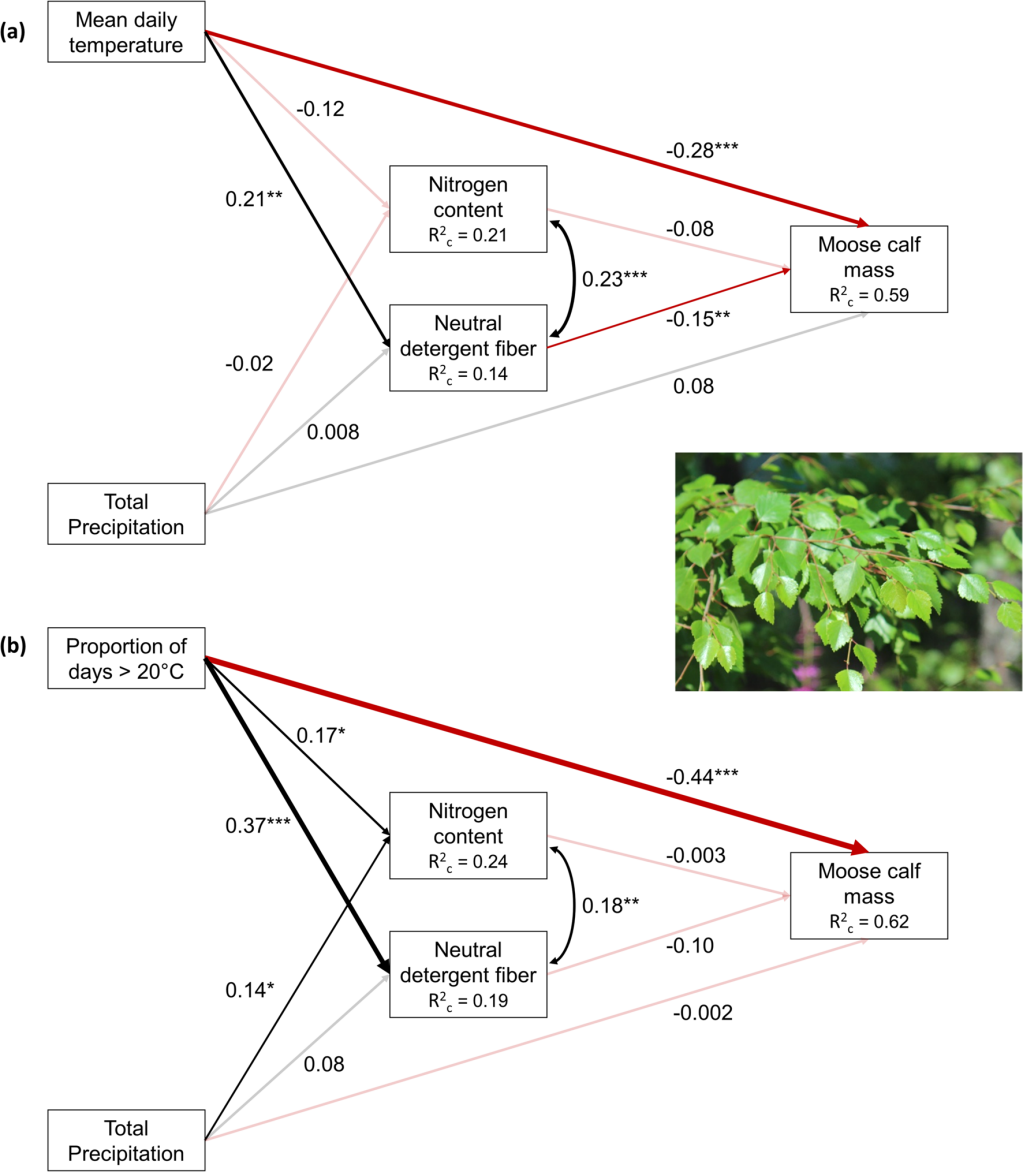
Results of the SEM comparing the direct relationship between weather, including **a** mean temperature and **b** proportion of days > 20 °C, and moose calf mass to the indirect relationship via nitrogen and neutral detergent fiber of birch leaves. Black lines represent positive relationships, red lines represent negative relationships. Line thickness is proportional to effect size. Transparent lines represent relationships with a *p *value more than the 0.05 significance level. R^2^_c_ values represent the variance of each dependent variable that is explained by fixed and random factors within that LMM

The sample size cutoffs imposed for the model limited our analyses to 32 sites (Fig. 1). The direct effects of temperature consistently showed stronger relationships to moose calf mass than did the indirect effects (Q1; Figs. 3, 4, supplementary materials Tables S3-S6)). When temperature was measured as the proportion of days with a maximum temperature of at least 20 °C, this direct relationship had a standardized estimate of greater magnitude than when temperature was measured as mean daily temperature (Q2). Temperature also had a stronger relationship with plant chemistry when measured as a proportion of days over 20 °C (Q2). However, while this temperature variable had the predicted negative relationship with N and positive relationship with NDF in fireweed (Fig. 3), in birch, both relationships were positive (Fig. 4). Precipitation showed a stronger relationship to plant chemistry for fireweed than for birch (Q3). The only indirect path with strong evidence to support it was between mean temperature, birch NDF, and calf mass. Higher mean growing season temperatures were associated with increased NDF, which was associated with lower calf mass (Q1; Fig. 4b).

## Discussion

### Direct vs. indirect effects

The direct effects of temperature consistently showed the strongest relationship to moose calf mass in our models (Q1; Figs. 3, 4). This suggests a direct impact of climate change on moose, through metabolic energy costs (McCann et al. 2013; Renecker and Hudson 1986, 1990), reduced feeding (Youngentob et al. 2021), and/or lost foraging opportunities while seeking thermal shelter (Van Beest et al. 2012). Adverse effects of high temperatures and the resulting heat stress have been reported for a number of other ruminant species. For example, Pérez-Barbería et al. (2020) found that heat stress reduced the growth rates of red deer (*Cervus elaphus*) calves and Semenzato et al. (2021) reported decreased activity and withdrawal into less suitable habitat for Alpine ibex (*Capra ibex*). Negative physiological effects of heat stress such as reduced fertility, declines in milk quantity and quality, and impairment of embryonic development have been well-documented in small domestic ruminants such as sheep and goats (reviewed in Al-Dawood 2017) but also larger ones such as cows (Dahl et al. 2019). Temperature effects can also act indirectly. For example, disruptions of feeding on warm days in reindeer (*Rangifer tarandus*) were found to be linked to insect harassment instead of thermal stress (Hagemoen and Reimers 2002). Models suggest that larger adult female moose are at greater risk of overheating more quickly than smaller individuals in less favorable environmental conditions (Verzuh et al. 2022), indicating that the impacts of direct heat stress may be greater for larger calves.

The only indirect path with moderate support suggests that higher mean temperatures are linked to increased NDF, which is, in turn, associated with lower calf mass (Q1; Fig. 4a). This supports the idea that with warmer spring/summers, phenology is advanced (Doi and Katano 2008; Douhard et al. 2018; Pettorelli et al. 2005), leading to an earlier peak in vegetation quality and also an earlier increase in fiber, and lower gain per unit food consumed during late spring or early summer (Albon and Langvatn 1992; Hebblewhite et al. 2008). However, contrary to predictions, we saw no positive impact of vegetation N on moose calf mass.

This could indicate that vegetation quality is not as singularly important in this environment as has been suggested. For example, while Herfindal and colleagues (2006) found a large effect of spring/summer forage quality on moose mass throughout Norway, they also linked years with earlier growing seasons to heavier calves and juveniles. In northern Sweden, moose parturition generally occurs prior to vegetation onset; therefore, energy intake may be limited more by the length of the short growing season (forage quantity) in this region (Neumann et al. 2020). It is likely that southern moose populations, in which parturition occurs after the start of the much longer growing season (Neumann et al. 2020), would show a stronger relationship between forage quality (in this case higher nitrogen, lower fiber) and moose calf mass. In this case, northern populations may be somewhat buffered against this potential indirect impact of rising temperatures. It is noteworthy that northern Sweden has not shown a pattern of declining calf mass over time, unlike more southern regions (Holmes et al. 2021).

Alternatively or additionally, herbivores like moose may be limited by other nutrients or macromolecules, in addition to N (Rizzuto et al. 2021). Moose may, therefore, use a nutrient balancing strategy, rather than protein maximization, as has been seen in winter studies (Felton et al. 2016; Felton et al. 2021; Spitzer et al. 2023). This could weaken the expected relationship between N concentration and moose calf mass.

Furthermore, maternal condition and maternal effects play an important role in calf mass (Cheynel et al. 2021). Thus, another important factor is that in capital breeders, like moose, a large proportion of the protein transferred from mother to calf during pregnancy and lactation likely comes from maternal stores, reflecting N in the previous late summer or fall vegetation, rather than that of the current year (Taillon et al. 2013). This means there could be a delayed impact of vegetation quality on calf mass, which we did not investigate here. Also, direct thermoregulatory costs to mothers could have indirect impacts on calves via milk nutrition or production during the lactation period. Sudden increases in summer temperature have been associated with a temporary drop in milk production in Murrah buffaloes, and were attributed to thermal stress (Upadhyay et al. 2007). Thus, direct effects of temperature on cows may influence calf mass indirectly via short- and long-term effects on females.

Population size may also influence calf mass as part of density-dependent population regulation, where one expects high population density to lead to reduced calf mass and vice versa for low population density (Taillon et al. 2012). The population density of moose has declined in Sweden from the early 1980s to the early 1990s, including in our study area, due to increased hunting pressure. The moose population in northern Sweden was then maintained at a relatively stable density until the new moose management was introduced in 2012, but has been reduced by 30% on average since then (Widemo et al. 2022; Älgdata, 2023). Declining calf mass, as in our study, could be explained by an increasing population size whereas we find the opposite during our study period. We, therefore, suggest that population size did not play a major role in determining changes in calf mass in our study.

While we observed relationships between temperature and both fiber and nitrogen content of forage, as seen in earlier studies (Bø and Hjeljord 1991; Lenart et al. 2002), the relationships were inconsistent, and may be linked to the number of days where the temperature exceeded 20 °C (or a similar threshold) rather than temperature in general. Precipitation appeared to play a larger role in fireweed forage quality than temperature, supporting findings that low water availability leads to lower plant protein content (Deléglise et al. 2015; White et al. 2014). While precipitation is predicted to increase overall in Sweden, this is concentrated in winter temporally, and in northern Sweden spatially, with southern locations being more likely to suffer dry conditions in summer, though there is variability across models (Belyazid and Zanchi 2019; IPCC 2021). This could have unpredictable impacts on moose forage in the future.

It is important to note that we only looked at two food species. While moose consume birch and fireweed heavily in some areas during the summer (Cederlund et al. 1980; Sæther et al. 1996), moose diets during the growing season are diverse and can vary over time (e.g., fireweed consumption spikes in August; Cederlund et al. 1980), across habitat types (Spitzer et al. 2020), with availability of different forage species (Wam and Hjeljord 2010), and with deer densities (Spitzer et al. 2021). It is, therefore, important to determine if other woody browse and herbaceous forage species (particularly those that make up a large proportion of the moose summer diet) show similar nutritional relationships with temperature and precipitation as do birch and fireweed. A preliminary study comparing changes in nutrient content in 12 tree and shrub species over 2 growing seasons—a heat wave and drought year, and the following year showed inconsistent results (Spitzer and Cromsigt 2021). The predominant trend for deciduous leaves and shrubs was a stronger increase in NDF during the drought year, though the reverse pattern was seen in deciduous twigs (Spitzer and Cromsigt 2021). However, this study did not include herbaceous vegetation (Spitzer and Cromsigt 2021). If patterns differ across forage species, moose may be able to maximize N intake by switching to foods that provide a greater concentration of N at any given time. Indeed, moose have been shown to balance macronutrient intake in winter through food item choice in both captive and wild studies (Felton et al. 2016; Felton et al. 2021; Spitzer et al. 2023).

### Average vs. extreme temperatures

The direct relationship between temperature and moose calf mass appeared to be more strongly linked to time above a biologically relevant threshold temperature (days with maximum temperature ≥ 20 °C; Q2) than to mean temperature. This further suggests that metabolic and/or behavioral thermoregulation costs are important for moose calves in their first summer. As heat extremes are predicted to become more frequent and intense over time (IPCC 2021), it may become even more important to monitor these thresholds when considering moose management in the future. This is particularly pertinent in these northern populations given the stronger direct relationship between temperature and calf mass compared to indirect paths. That said, fixed maximum temperature thresholds are more likely to be crossed in warmer regions, indicating the potential for even greater impact in southern Sweden. Temperature also had a stronger relationship with plant chemistry when measured as a proportion of days over 20 °C (Q2), though this did not translate into stronger indirect impacts on calf mass.

### Vegetation type

Precipitation showed a stronger relationship to plant chemistry for fireweed than for birch (Q3). This partially supports the hypothesis that weather is more relevant for annuals/forbs. However, our results did not suggest a larger effect of temperature on the advance of spring phenology (at least with respect to the potential chemical indicators of N and NDF) in annuals than perennials, as has been suggested in other studies (Stuble et al. 2021). Additionally, fireweed quality did not appear to have as strong a relationship to calf mass as did birch.

### Interactions

While the number of ≥ 20 °C days had the predicted negative relationship with N and positive relationship with NDF in fireweed (Fig. 3), in birch, both relationships were positive, and the correlation between N and NDF was also unexpectedly positive (Fig. 4). This could indicate an unexplored factor in our models, or possibly an interaction between temperature and water availability, with trees or shrubs able to access deeper water sources compared to herbaceous vegetation when water is scarce (e.g., Darrouzet-Nardi et al. 2006; Kulmatiski and Beard 2013). It would be important to investigate if these patterns hold across annual vs. perennial species more broadly.

There is an apparent interaction between temperature and precipitation in their relationship to moose calf mass in Sweden, with high temperatures associated with lighter calves when May/June precipitation is below average, but heavier calves with high levels of precipitation (Holmes et al. 2021). It is possible that this interaction will play a strong role in indirect effects of forage quantity, as plant growth and productivity have been shown to increase with both warming and heavy precipitation but decrease with warming and dry conditions (Hoeppner and Dukes 2012; Wu et al. 2011). Long-term monitoring of the impacts of temperature and precipitation on forage quantity and quality are, thus, recommended, particularly for northern regions where quantity may play a larger role in moose nutrition (see “Direct vs. indirect effects”, above).

### Caveats

An additional potential explanation for the unexpected relationships between N and NDF and lack of strong indirect pathways found in this study could be linked to the way that NIRS-based models were built. There was a good to excellent relationship between predicted and observed for all three variables (dry matter, NDF, N; Supplementary Figs. 2S and 3S). Ideally, however, this relationship should be tested on an independent dataset. This was not possible in this study due to a limited number of samples available for model calibration. Another caveat is that we measured variation in food quality for a long time period but only at a given time period during the middle of summer and not throughout the season. Moose calf weights would not be determined by the food quality at only that given time but by the quality throughout a longer period of spring and/or summer. In our study, we are, thus, using the snapshot of the forage quality during the middle of summer as a measure for the general trend in phenological progression that year (i.e., a low value during the middle of summer would indicate a year where quality had already started to decline and would unlikely increase substantially later on in the summer). We also sampled during the middle of summer as an important period during calf growth, when the quality and quantity of forage will have influenced calf mass indirectly both by limiting the cows’ ability to produce milk and through effects on forage consumed by calves. Future studies should look into the importance of long-term trends in within-season variation of food quality.

## Conclusion

Environmental change can impact organisms in multiple ways, both direct and indirect, and these effects can be difficult to disentangle. However, by attempting to isolate different causal pathways, we can learn which risks are strongest or most imminent and can better focus mitigation efforts. In this case, while indirect impacts of climate change on forage deserve further investigation, it is vital to recognize the large direct impacts of temperature on cold-adapted species, including moose. Likewise, rather than simply identifying broad trends with environmental averages, identifying which aspects of environmental change are most tightly linked to consequences can improve risk assessment precision, and help target ways to interrupt pathways to impact. Here, we show that it may be more relevant to monitor the occurrence of temperature extremes than mean trends when determining the potential impacts of climate change on moose. Finally, it is important to consider potential differences in the effects of climate on annual vs. perennial food plants, as well as the impacts of these plants on the organisms that consume them. Diet shifting, nutrient balancing, and other forms of behavioral flexibility may yet play a large role in alleviative responses to climate change.

## Supplementary Information

Below is the link to the electronic supplementary material.

Supplementary file1 (DOCX 2007 KB)

## Data Availability

The prepared dataset used in the piecewise SEM can be found at https://doi.org/10.5878/j1fh-8w11.

## Code availability

Code for the piecewise SEM can be found at https://doi.org/10.5878/j1fh-8w11.

## References

[CR1] Albon SD, Langvatn R (1992). Plant phenology and the benefits of migration in a temperate ungulate. Oikos.

[CR2] Albon SD, Clutton-Brock TH, Guinness FE (1987). Early development and population dynamics in red deer ii density-independent effects and cohort variation. J Animal Ecol..

[CR3] Al-Dawood A (2017). Towards heat stress management in small ruminants-a review. Ann Animal Sci.

[CR4] Ball JP, Danell K, Sunesson P (2000). Response of a herbivore community to increased food quality and quantity: an experiment with nitrogen fertilizer in a boreal forest. J Appl Ecol.

[CR5] Beard KH, Kelsey KC, Leffler AJ, Welker JM (2019). The missing angle: ecosystem consequences of phenological mismatch. Trends Ecol Evol.

[CR6] Belyazid S, Zanchi G (2019). Water limitation can negate the effect of higher temperatures on forest carbon sequestration. Eur J Forest Res.

[CR7] Beniston M (2007). Future extreme events in European climate: an exploration of regional climate model projections. Clim Change.

[CR8] Bø S, Hjeljord O (1991). Do continental moose ranges improve during cloudy summers?. Can J Zool.

[CR9] Capoani L, Capoani L (2019). Variations in nutritional content of key ungulate browse species in Sweden. Master Master's thesis, Swedish University of Agricultural Sciences.

[CR10] Cederlund G, Ljungqvist H, Markgren G (1980). Foods of moose and roe-deer at Grimsö in central Sweden - results of rumen content analyses. Swed Wildlife Res Viltrevy.

[CR11] Cheynel L (2021). Maternal effects shape offspring physiological condition but do not senesce in a wild mammal. J Evol Biol.

[CR12] Christianson D, Creel S (2009). Effects of grass and browse consumption on the winter mass dynamics of elk. Oecologia.

[CR13] Cohen JM, Lajeunesse MJ, Rohr JR (2018). A global synthesis of animal phenological responses to climate change. Nat Clim Chang.

[CR14] Dahl GE, Skibiel AL, Laporta J (2019). In utero heat stress programs reduced performance and health in calves. Veter Clin North Amer Food Ani Pract.

[CR15] Darrouzet-Nardi A, D’Antonio CM, Dawson TE (2006). Depth of water acquisition by invading shrubs and resident herbs in a Sierra Nevada meadow. Plant Soil.

[CR16] Deléglise C (2015). Drought-induced shifts in plants traits, yields and nutritive value under realistic grazing and mowing managements in a mountain grassland. Agr Ecosyst Environ.

[CR17] Ditmer MA, Moen RA, Windels SK, Forester JD, Ness TE, Harris TR (2018). Moose at their bioclimatic edge alter their behavior based on weather, landscape, and predators. Current Zool.

[CR18] Doi H, Katano I (2008). Phenological timings of leaf budburst with climate change in Japan. Agri for Meteorol..

[CR19] Dou H, Jiang G, Stott P, Piao R (2013). Climate change impacts population dynamics and distribution shift of moose (Alces alces) in Heilongjiang Province of China. Ecol Res.

[CR20] Douhard M, Guillemette S, Festa-Bianchet M, Pelletier F (2018). Drivers and demographic consequences of seasonal mass changes in an alpine ungulate. Ecology.

[CR21] Felton AM (2016). The nutritional balancing act of a large herbivore: an experiment with captive moose (Alces alces L). PLoS ONE.

[CR22] Felton AM (2021). Macronutrient balancing in free-ranging populations of moose. Ecol Evol.

[CR23] Festa-Bianchet M, Jorgenson JT, Bérubé CH, Portier C, Wishart WD (1997). Body mass and survival of bighorn sheep. Can J Zool.

[CR24] Forsyth D, Richardson S, Menchenton K (2005). Foliar fibre predicts diet selection by invasive red deer Cervus elaphus scoticus in a temperate New Zealand forest. Funct Ecol.

[CR25] Fuller A, Mitchell D, Maloney SK, Hetem RS (2016). Towards a mechanistic understanding of the responses of large terrestrial mammals to heat and aridity associated with climate change. Clim Change Resp.

[CR26] Gaillard J-M, Loison A, Toïgo C, Delorme D, Van Laere G (2003). Cohort effects and deer population dynamics. Écoscience.

[CR27] Hagemoen RIM, Reimers E (2002). Reindeer summer activity pattern in relation to weather and insect harassment. J Anim Ecol.

[CR28] Hebblewhite M, Merrill E, McDermid G (2008). A multi-scale test of the forage maturation hypothesis in a partially migratory ungulate population. Ecol Monogr.

[CR29] Herfindal I, Sæther B-E, Solberg EJ, Andersen R, Høgda KA (2006). Population characteristics predict responses in moose body mass to temporal variation in the environment. J Anim Ecol.

[CR30] Hetem RS, Fuller A, Maloney SK, Mitchell D (2014). Responses of large mammals to climate change. Temperature.

[CR31] Hoeppner SS, Dukes JS (2012). Interactive responses of old-field plant growth and composition to warming and precipitation. Glob Change Biol.

[CR32] Holmes SM, Cromsigt JP, Danell K, Ericsson G, Singh NJ, Widemo F (2021). Declining recruitment and mass of Swedish moose calves linked to hot, dry springs and snowy winters. Global Ecol Conserv.

[CR33] IPCC (2021) Climate Change 2021: The Physical Science Basis. Contribution of Working Group I to the Sixth Assessment Report of the Intergovernmental Panel on Climate Change. In: Masson-Delmotte V, (eds), Cambridge University Press, Cambridge, United Kingdom and New York, NY, USA

[CR34] Jarvis P, Linder S (2000). Constraints to growth of boreal forests. Nature.

[CR35] Jones H (2019). Mortality assessment of moose (Alces alces) calves during successive years of winter tick (Dermacentor albipictus) epizootics in New Hampshire and Maine (USA). Can J Zool.

[CR36] Keech MA (2011). Effects of predator treatments, individual traits, and environment on moose survival in Alaska. J Wildl Manag.

[CR37] Kulmatiski A, Beard KH (2013). Root niche partitioning among grasses, saplings, and trees measured using a tracer technique. Oecologia.

[CR38] Langvatn R, Langvatn R (1977). Criteria of physical condition, growth and development in Cervidae - suitable for routine studies. Nordic Council for Wildlife Research.

[CR39] Lefcheck JS (2016). piecewiseSEM: Piecewise structural equation modelling in r for ecology, evolution, and systematics. Methods Ecol Evol.

[CR40] Lenart EA, Bowyer RT, Hoef JV, Ruess RW (2002). Climate change and caribou: effects of summer weather on forage. Can J Zool.

[CR41] Liland KH, Mevik B-H, Wehrens R (2021) pls: Partial Least Squares and Principal Component Regression

[CR42] Loison A, Jullien J-M, Menaut P (1999). Relationship between chamois and isard survival and variation in global and local climate regimes: contrasting examples from the Alps and Pyrenees. Ecol Bullet..

[CR43] Månsson J, Bergström R, Danell K (2009). Fertilization—effects on deciduous tree growth and browsing by moose. For Ecol Manage.

[CR44] McArt SH, Spalinger DE, Collins WB, Schoen ER, Stevenson T, Bucho M (2009). Summer dietary nitrogen availability as a potential bottom-up constraint on moose in south-central Alaska. Ecology.

[CR45] McCann N, Moen R, Harris T (2013). Warm-season heat stress in moose (Alces alces). Can J Zool.

[CR46] McKechnie AE, Wolf BO (2019). The physiology of heat tolerance in small endotherms. Physiology.

[CR47] Meehl GA (2000). An introduction to trends in extreme weather and climate events: observations, socioeconomic impacts, terrestrial ecological impacts, and model projections. Bull Am Meteor Soc.

[CR48] Monteith KL, Klaver RW, Hersey KR, Holland AA, Thomas TP, Kauffman MJ (2015). Effects of climate and plant phenology on recruitment of moose at the southern extent of their range. Oecologia.

[CR49] Murray DL (2006). Pathogens, nutritional deficiency, and climate influences on a declining moose population. Wildl Monogr.

[CR50] Neumann W (2020). Divergence in parturition timing and vegetation onset in a large herbivore—differences along a latitudinal gradient. Biol Let.

[CR51] Olff H, Ritchie ME, Prins HHT (2002). Global environmental controls of diversity in large herbivores. Nature.

[CR52] Parker KL, Robbins CT (1984). Thermoregulation in mule deer and elk. Can J Zool.

[CR53] Parker KL, Barboza PS, Gillingham MP (2009). Nutrition integrates environmental responses of ungulates. Funct Ecol.

[CR54] Peltier TC, Barboza PS (2003). Growth in an arctic grazer: effects of sex and dietary nitrogen on yearling muskoxen. J Mammal.

[CR55] Pérez-Barbería FJ, García AJ, Cappelli J, Landete-Castillejos T, Serrano MP, Gallego L (2020). Heat stress reduces growth rate of red deer calf: Climate warming implications. PLoS ONE.

[CR56] Pettorelli N, Pelletier F, Hardenberg AV, Festa-Bianchet M, CôTé SD (2007). Early onset of vegetation growth vs rapid green-up: impacts on juvenile mountain ungulates. Ecology.

[CR57] Pettorelli N, Mysterud A, Yoccoz NG, Langvatn R, Stenseth NC (2005) Importance of climatological downscaling and plant phenology for red deer in heterogeneous landscapes Proceedings of the Royal Society of London. Series B Biological Sciences. 272:2357–2364. Doi: 10.1098/rspb.2005.3218 10.1098/rspb.2005.3218 PMC1559971 16243701

[CR58] Pinheiro J, Bates D, R Core Team (2022) nlme: Linear and Nonlinear Mixed Effects Models

[CR59] Plard F (2014). Mismatch between birth date and vegetation phenology slows the demography of roe deer. PLoS Biol.

[CR60] Post E, Forchhammer MC (2008). Climate change reduces reproductive success of an Arctic herbivore through trophic mismatch. Phil Trans Royal Soci Biol Sci.

[CR61] Price DT (2013). Anticipating the consequences of climate change for Canada’s boreal forest ecosystems. Environ Rev.

[CR62] Rempel RS (2011). Effects of climate change on moose populations: exploring the response horizon through biometric and systems models. Ecol Model.

[CR63] Renecker LA, Hudson RJ (1986). Seasonal energy expenditures and thermoregulatory responses of moose. Can J Zool.

[CR64] Renecker LA, Hudson RJ (1990). Behavioral and thermoregulatory responses of moose to high ambient temperatures and insect harassment in aspen-dominated forests. Alces J Devot Biol Manag Moose..

[CR65] Rizzuto M (2021). Forage stoichiometry predicts the home range size of a small terrestrial herbivore. Oecologia.

[CR66] Rondinini C, Visconti P (2015). Scenarios of large mammal loss in Europe for the 21st century. Conserv Biol.

[CR67] Sæther B-E, Andersen R, Hjeljord O, Heim M (1996). Ecological correlates of regional variation in life history of the moose alces alces. Ecology.

[CR68] Schrempp TV (2019). Linking forest management to moose population trends: The role of the nutritional landscape. PLoS ONE.

[CR69] Semenzato P (2021). Behavioural heat-stress compensation in a cold-adapted ungulate: Forage-mediated responses to warming Alpine summers. Ecol Lett.

[CR70] Shipley B (2009). Confirmatory path analysis in a generalized multilevel context. Ecology.

[CR71] Shively RD, Crouse JA, Thompson DP, Barboza PS (2019). Is summer food intake a limiting factor for boreal browsers? Diet, temperature, and reproduction as drivers of consumption in female moose. PLoS ONE.

[CR72] Spalinger DE, Collins W, Hanley TA, Cassara N, Carnahan A (2010). The impact of tannins on protein, dry matter, and energy digestion in moose (Alces alces). Can J Zool.

[CR73] Spitzer R, Felton A, Landman M, Singh NJ, Widemo F, Cromsigt JP (2020). Fifty years of European ungulate dietary studies: a synthesis. Oikos.

[CR74] Spitzer R (2021). Small shrubs with large importance? Smaller deer may increase the moose-forestry conflict through feeding competition over Vaccinium shrubs in the field layer. For Ecol Manage.

[CR75] Spitzer R (2023). Macro-nutritional balancing in a circumpolar boreal ruminant under winter conditions. Funct Ecol.

[CR76] Spitzer R, Cromsigt JP (2021) Effects of summer drought on ungulate forage quality and habitat selection in Northern Sweden

[CR77] Stuble KL, Bennion LD, Kuebbing SE (2021). Plant phenological responses to experimental warming—A synthesis. Glob Change Biol.

[CR78] Taillon J, Brodeur V, Festa-Bianchet M, Côté S (2012). Is mother condition related to offspring condition in migratory caribou (Rangifer tarandus) at calving and weaning?. Can J Zool.

[CR79] Taillon J, Barboza PS, Côté SD (2013). Nitrogen allocation to offspring and milk production in a capital breeder. Ecology.

[CR80] Thompson DP (2021). Behaviour influences thermoregulation of boreal moose during the warm season. Conserv Physiol..

[CR81] Upadhyay R, Singh S, Kumar A, Gupta S (2007). Impact of climate change on milk production of Murrah buffaloes. Ital J Ani Sci.

[CR82] Van Beest FM, Milner JM (2013). Behavioural responses to thermal conditions affect seasonal mass change in a heat-sensitive northern ungulate. PLoS ONE.

[CR83] Van Beest FM, Van Moorter B, Milner JM (2012). Temperature-mediated habitat use and selection by a heat-sensitive northern ungulate. Anim Behav.

[CR84] Verzuh TL, Hall LE, Cufaude T, Knox L, Class C, Monteith KL (2021). Behavioural flexibility in a heat-sensitive endotherm: the role of bed sites as thermal refuges. Anim Behav.

[CR85] Verzuh TL (2022). Behavioral responses of a large, heat-sensitive mammal to climatic variation at multiple spatial scales. J Ani Ecol..

[CR86] Wam HK, Hjeljord O (2010). Moose summer and winter diets along a large scale gradient of forage availability in southern Norway. Eur J Wildl Res.

[CR87] Weiskopf SR, Ledee OE, Thompson LM (2019). Climate change effects on deer and moose in the Midwest. J Wildl Manag.

[CR88] White RG (1983). Foraging patterns and their multiplier effects on productivity of northern ungulates. Oikos.

[CR89] White SR, Cahill JF, Bork EW (2014). Implications of precipitation, warming, and clipping for grazing resources in Canadian prairies. Agron J.

[CR90] Widemo F, Leonardsson K, Ericsson G (2022) Samförvaltning avälg och skog–analyser av den nya älgförvaltningen under perioden 2012–2021

[CR91] Wu Z, Dijkstra P, Koch GW, Peñuelas J, Hungate BA (2011). Responses of terrestrial ecosystems to temperature and precipitation change: a meta-analysis of experimental manipulation. Glob Change Biol.

[CR92] Youngentob KN, Lindenmayer DB, Marsh KJ, Krockenberger AK, Foley WJ (2021). Food intake: an overlooked driver of climate change casualties?. Trends Ecol Evol.

