# Supplementary material for: Increased summer temperature is associated with reduced calf mass of a circumpolar large mammal through direct thermoregulatory and indirect, food quality, pathways
Source: Oecologia. 2023 Apr 5;201(4):1123–36. doi: 10.1007/s00442-023-05367-0 (PMC10113315; doi:10.1007/s00442-023-05367-0)
Supplement: Supplementary file 1 — Supplementary file1 (DOCX 2007 KB) [file 442_2023_5367_MOESM1_ESM.docx]

**SUPPLEMENTARY MATERIALS**

Table 1S. The number of hunting teams and number of calf slaughter weights reported in each year, from 1988 to 1997.

| Year | Number of teams | Number of weights reported | |
| --- | --- | --- | --- |
| 1988 | 117 | 1224 |  |
| 1989 | 226 | 1680 |  |
| 1990 | 145 | 1262 |  |
| 1991 | 120 | 991 |  |
| 1992 | 203 | 1196 |  |
| 1993 | 308 | 1374 |  |
| 1994 | 309 | 1407 |  |
| 1995 | 329 | 1356 |  |
| 1996^1^ | 247 | 1140 |  |
| 1997 | 228 | 1117 |  |

^1^14 calf weights were not associated with any team in 1996

Table 2S. Leave-one-out cross validation performance metrics of the partial least square regression for the dry matter (DM), neutral detergent fibre (NDF) and nitrogen (N) contents.

| variable | rmse | rrmse (%) | r2 |
| --- | --- | --- | --- |
| DM | 0.366 | 0.42 | 0.63 |
| NDF | 2.67 | 7.7 | 0.69 |
| N | 0.157 | 7.07 | 0.88 |

Table 3S. Results of the SEM comparing the direct relationship between weather and mean moose calf mass to the indirect relationship via nitrogen and neutral detergent fiber of fireweed stems, leaves, and flowers. Temperature was measured as mean daily temperature.

| Response | Predictor | Estimate | SE | p | Standardized Estimate |  |
| --- | --- | --- | --- | --- | --- | --- |
| NDF | Temperature | 0.256 | 0.243 | 0.292 | 0.069 |  |
| NDF | Precipitation | -0.063 | 0.008 | <0.001 | -0.526 | *** |
| N | Temperature | -0.030 | 0.021 | 0.154 | -0.101 |  |
| N | Precipitation | 0.003 | 0.001 | <0.001 | 0.347 | *** |
| Calf Weight | Temperature | -1.053 | 0.244 | <0.001 | -0.283 | *** |
| Calf Weight | Precipitation | 0.009 | 0.009 | 0.318 | 0.074 |  |
| Calf Weight | N | -1.224 | 0.957 | 0.202 | -0.097 |  |
| Calf Weight | NDF | -0.093 | 0.081 | 0.251 | -0.093 |  |
| ~~NDF | ~~N | -0.609 | - | <0.001 | -0.609 | *** |

Table 4S. Results of the SEM comparing the direct relationship between weather and mean moose calf mass to the indirect relationship via nitrogen (N) and neutral detergent fiber (NDF) of fireweed stems, leaves, and flowers. Temperature was measured as the proportion of days where the temperature exceeded 20 °C.

| Response | Predictor | Estimate | SE | p | Standardized Estimate |  |
| --- | --- | --- | --- | --- | --- | --- |
| NDF | Temperature | 5.123 | 2.528 | 0.044 | 0.126 | * |
| NDF | Precipitation | -0.060 | 0.007 | <0.001 | -0.498 | *** |
| N | Temperature | -0.460 | 0.219 | 0.037 | -0.143 | * |
| N | Precipitation | 0.003 | 0.001 | <0.001 | 0.326 | *** |
| Calf Weight | Temperature | -19.339 | 2.345 | <0.001 | -0.473 | *** |
| Calf Weight | Precipitation | -0.001 | 0.008 | 0.881 | -0.010 |  |
| Calf Weight | N | -1.685 | 0.897 | 0.062 | -0.133 |  |
| Calf Weight | NDF | -0.088 | 0.075 | 0.237 | -0.088 |  |
| ~~NDF | ~~N | -0.606 | - | <0.001 | -0.606 | *** |

Table 5S. Results of the SEM comparing the direct relationship between weather and mean moose calf mass to the indirect relationship via nitrogen and neutral detergent fiber of birch leaves. Temperature was measured as mean daily temperature.

| Response | Predictor | Estimate | SE | p | Standardized Estimate |  |
| --- | --- | --- | --- | --- | --- | --- |
| NDF | Temperature | 0.553 | 0.200 | 0.006 | 0.210 | ** |
| NDF | Precipitation | 0.001 | 0.006 | 0.912 | 0.008 |  |
| N | Temperature | -0.034 | 0.020 | 0.099 | -0.120 |  |
| N | Precipitation | 0.000 | 0.001 | 0.744 | -0.022 |  |
| Calf Weight | Temperature | -1.034 | 0.245 | <0.001 | -0.277 | *** |
| Calf Weight | Precipitation | 0.009 | 0.008 | 0.213 | 0.078 |  |
| Calf Weight | N | -1.080 | 0.793 | 0.175 | -0.081 |  |
| Calf Weight | NDF | -0.219 | 0.080 | 0.007 | -0.154 | ** |
| ~~NDF | ~~N | 0.234 | - | <0.001 | 0.234 | *** |

Table 6S. Results of the SEM comparing the direct relationship between weather and mean moose calf mass to the indirect relationship via nitrogen and neutral detergent fiber of birch leaves. Temperature was measured as the proportion of days where the temperature exceeded 20 °C.

| Response | Predictor | Estimate | SE | p | Standardized Estimate |  |
| --- | --- | --- | --- | --- | --- | --- |
| NDF | Temperature | 10.656 | 1.967 | <0.001 | 0.369 | *** |
| NDF | Precipitation | 0.007 | 0.006 | 0.233 | 0.080 |  |
| N | Temperature | 0.513 | 0.201 | 0.012 | 0.167 | * |
| N | Precipitation | 0.001 | 0.001 | 0.025 | 0.144 | * |
| Calf Weight | Temperature | -17.840 | 2.461 | <0.001 | -0.436 | *** |
| Calf Weight | Precipitation | 0.000 | 0.007 | 0.972 | -0.002 |  |
| Calf Weight | N | -0.044 | 0.750 | 0.953 | -0.003 |  |
| Calf Weight | NDF | -0.144 | 0.078 | 0.065 | -0.102 |  |
| ~~NDF | ~~N | 0.181 | - | 0.003 | 0.181 | ** |


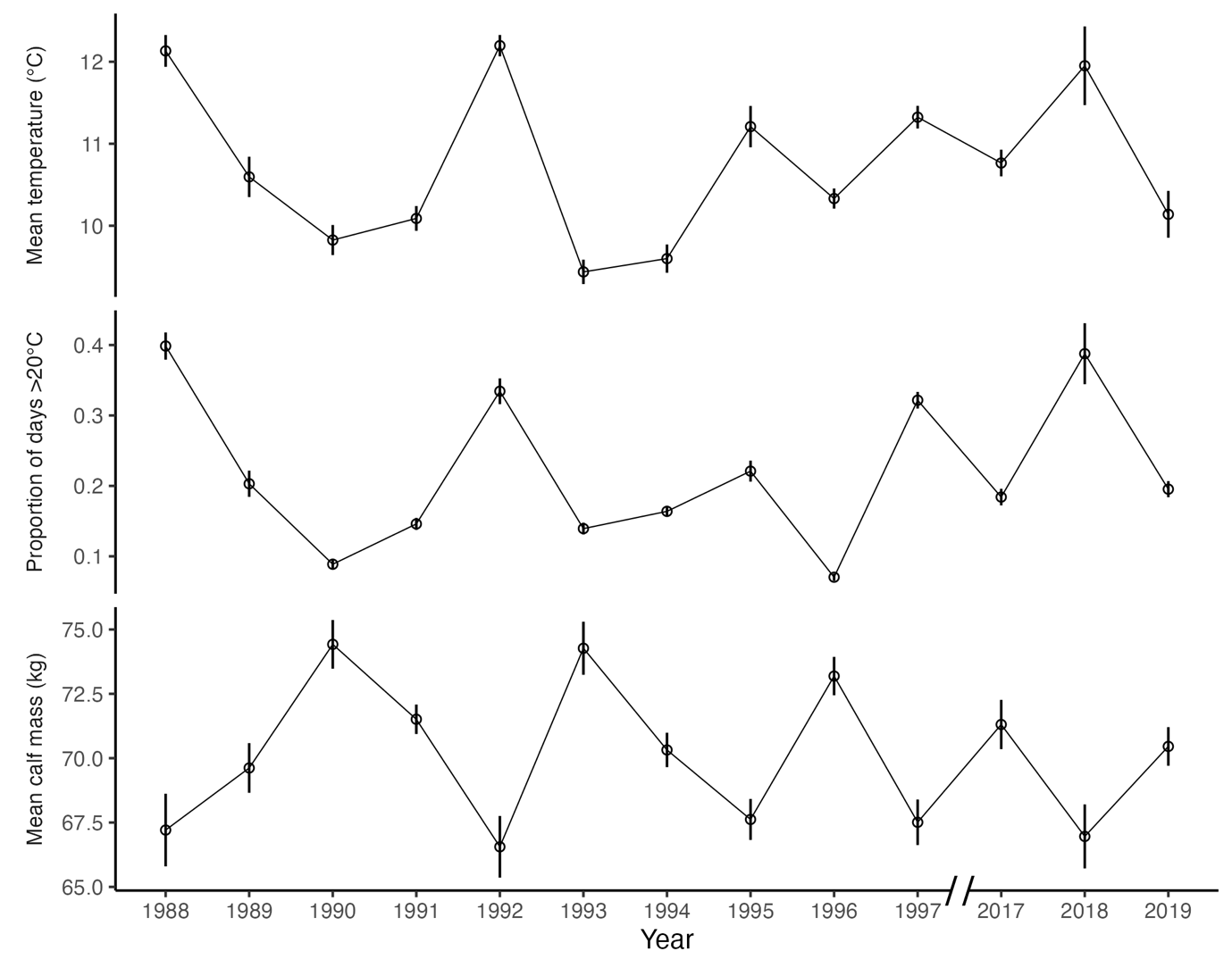


Fig. 1S Variation in mean temperature during the growing season, the proportion of hot days (≥ 20°C), and mean calf mass in the study area for the years included in the study. Values are presented as means across the study sites with error bars denoting the standard error.


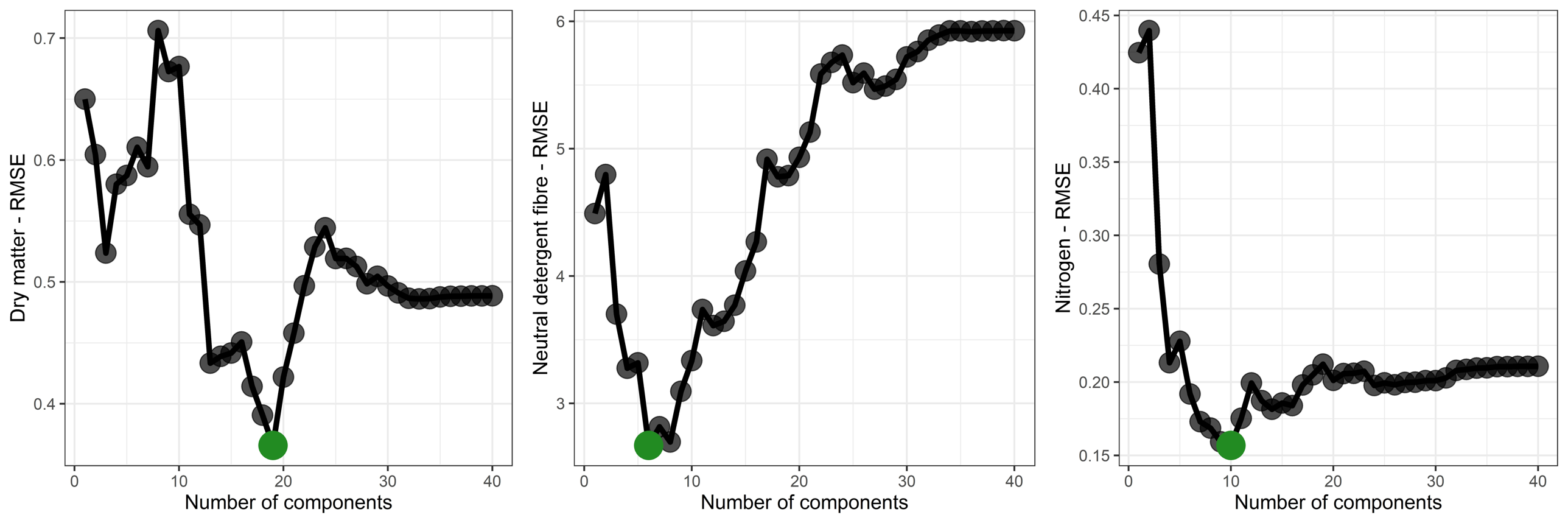


Figure 2S. Selection of the optimal number of components of the partial least square models for each variable of interest based on the RMSE of the leave-one-out cross validation.


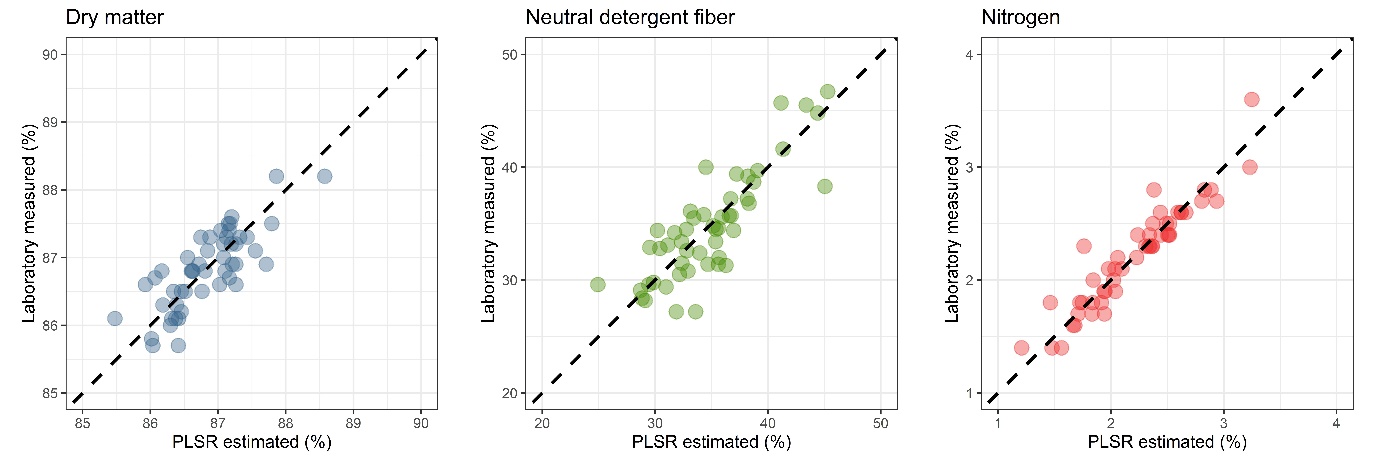


Figure 3S. Scatterplots of the partial least square-estimated vs laboratory measured values of the variables of interest. The dashed black line indicates the 1:1 regression line.
